# Supplementary material for: Phenolic Compound Profiles, Cytotoxic, Antioxidant, Antimicrobial Potentials and Molecular Docking Studies of Astragalus gymnolobus Methanolic Extracts
Source: Plants (Basel). 2024 Feb 27;13(5):658. doi: 10.3390/plants13050658 (PMC10934167; doi:10.3390/plants13050658)
Supplement: Supplementary file 1 [file plants-13-00658-s001.zip › plants-2876417-supplementary.pdf]

Supplementary File

Phenolic Compound Profiles, Cytotoxic, Antioxidant, Antimicrobial Potentials and Molecular Docking Studies of *Astragalus gymnolobus* Methanolic Extracts

Esra Aydemir [1,*], Elif Odabaş Köse [2], Mustafa Yavuz [1], A. Cansu Kilit [1], Alaaddin Korkut [1], Serap Özkaya Gül [1], Cengiz Sarikurkcu [3], Mehmet Engin Celep [4] and R. Süleyman Göktürk [1]

[1]Department of Biology, Faculty of Science, Akdeniz University, TR-07058 Antalya, Turkey; myavuz@akdeniz.edu.tr (M.Y.); cansukilit@akdeniz.edu.tr (A.C.K.); 202151005003@ogr.akdeniz.edu.tr (A.K.); 202151006001@ogr.akdeniz.edu.tr (S.Ö.G.); gokturk@akdeniz.edu.tr (R.S.G.)
[2]Medical Laboratory Program, Vocational School of Health Services, Akdeniz University, TR-07058 Antalya, Turkey; elifkose@akdeniz.edu.tr
[3]Department of Analytical Chemistry, Faculty of Pharmacy, Afyonkarahisar Health Sciences University,
TR-03100 Afyonkarahisar, Turkey; cengiz.sarikurkcu@afsu.edu.tr
[4]Department of Pharmacognosy, Faculty of Pharmacy, Yeditepe University, Atasehir, TR-34755 Istanbul, Turkey; ecelep@yeditepe.edu.tr
*Correspondence: esra@akdeniz.edu.tr

1. Phytochemical analysis

A simple, rapid, reproducible, and sensitive method, which was previously developed and validated, was used for the simultaneous determination of 30 phenolic compounds using LC–ESI–MS/MS. An Agilent Technologies 1260 Infinity liquid chromatography system hyphenated to a 6420 Triple Quad mass spectrometer was used for quantitative analyses. Chromatographic separation was carried out on a Poroshell 120 EC-C18 (100 mm × 4.6 mm I.D., 2.7 µm) column. The mobile phase configuration (0.1% formic acid/methanol) was selected on the base of the better chromatographic resolution of isomeric compounds. On the other hand, the selected mobile phase configuration also provided higher sensitivity for many of the phenolic compounds. As a result, the mobile phase was made up from solvent A (0.1%, *v*/*v* formic acid solution) and solvent B (methanol). The gradient profile was set as follows: 0.00 min 2% B eluent, 3.00 min 2% B eluent, 6.00 min 25% B eluent, 10.00 min 50% B eluent, 14.00 min 95% B eluent, 17.00 min 95% B and 17.50 min 2% B eluent. The column temperature was maintained at 25°C. The flow rate was 0.4 mL min^{-1} and the injection volume was 2.0 µL. The tandem mass spectrometer was interfaced to the LC system via an ESI source. The electrospray source of the MS was operated in negative and positive multiple reaction monitoring (MRM) mode and the interface conditions were as follows: capillary voltage of −3.5 kV, gas temperature of 300°C and gas flow of 11 L min^{-1}. The nebulizer pressure was 40 psi.

In negative and positive multiple reaction monitoring (MRM) mode, the peaks of the analytes were identified by comparing the retention time, together with the monitoring ions pairs in an authentic standard solution.

Table S1. ESI–MS/MS Parameters and analytical characteristics for the Analysis of Target Analytes by MRM Negative and Positive Ionization Mode

Target compounds	Rt (min)	Precursor ion	MRM1 (CE, V)	MRM2 (CE, V)
Compounds analyzed by NI mode				
Gallic acid	8.891	168.9 [M − H]−	125.0 (10)	–
Protocatechuic acid	10.818	152.9 [M − H]−	108.9 (12)	–
3,4-Dihydroxyphenylacetic acid	11.224	167.0 [M − H]−	123.0 (2)	–
(+)-Catechin	11.369	289.0 [M − H]−	245.0 (6)	202.9 (12)
Pyrocatechol	11.506	109.0 [M − H]−	90.6 (18)	52.9 (16)
2,5-Dihydroxybenzoic acid	12.412	152.9 [M − H]−	109.0 (10)	–
4-Hydroxybenzoic acid	12.439	136.9 [M − H]−	93.1 (14)	–
Caffeic acid	12.841	179.0 [M − H]−	135.0 (12)	–
Syringic acid	12.963	196.9 [M − H]−	181.9 (8)	152.8 (6)
3-Hydroxybenzoic acid	13.259	137.0 [M − H]−	93.0 (6)	–
Vanillin	13.397	151.0 [M − H]−	136.0 (10)	–
Verbascoside	13.589	623.0 [M − H]−	461.0 (26)	160.8 (36)
Taxifolin	13.909	303.0 [M − H]−	285.1 (2)	125.0 (14)
Sinapic acid	13.992	222.9 [M − H]−	207.9 (6)	163.8 (6)
p-Coumaric acid	14.022	162.9 [M − H]−	119.0 (12)	–
Ferulic acid	14.120	193.0 [M − H]−	177.8 (8)	134.0 (12)
Luteolin 7-glucoside	14.266	447.1 [M − H]−	285.0 (24)	–
Rosmarinic acid	14.600	359.0 [M − H]−	196.9 (10)	160.9 (10)
2-Hydroxycinnamic acid	15.031	162.9 [M − H]−	119.1 (10)	–
Pinoresinol	15.118	357.0 [M − H]−	151.0 (12)	135.7 (34)
Eriodictyol	15.247	287.0 [M − H]−	151.0 (4)	134.9 (22)
Quercetin	15.668	301.0 [M − H]−	178.6 (10)	151.0 (16)
Kaempferol	16.236	285.0 [M − H]−	242.8 (16)	229.1 (18)
Compounds analyzed by PI mode				
Chlorogenic acid	11.802	355.0 [M + H]+	163.0 (10)	–
(−)-Epicatechin	12.458	291.0 [M + H]+	139.1 (12)	122.9 (36)
Hesperidin	14.412	611.1 [M + H]+	449.2 (4)	303.0 (20)
Hyperoside	14.506	465.1 [M + H]+	303.1 (8)	–
Apigenin 7-glucoside	14.781	433.1 [M + H]+	271.0 (18)	–
Luteolin	15.923	287.0 [M + H]+	153.1 (34)	135.1 (36)
Apigenin	16.382	271.0 [M + H]+	153.0 (34)	119.1 (36)

R_t, retention time; NI, negative ion; and PI, positive ion.

Table S2. Calibration curves and sensitivity properties of the method

Compounds	Linearity and sensitivity characteristics				
	Range (μg/L)	Linear equation	R2	LOD (μg/L)	LOQ (μg/L)
Gallic acid	5–500	y = 4.82x − 26.48	0.9988	1.46	4.88
Protocatechuic acid	2.5–500	y = 5.65x − 9.99	0.9990	1.17	3.88
3,4-Dihydroxyphenylacetic acid	5–500	y = 5.13x − 12.39	0.9990	1.35	4.51
(+)-Catechin	10–500	y = 1.45x + 1.95	0.9974	3.96	13.20
Pyrocatechol	25–400	y = 0.11x − 0.52	0.9916	9.62	32.08
Chlorogenic acid	1–500	y = 12.14x + 32.34	0.9995	0.55	1.82
2,5-Dihydroxybenzoic acid	5–500	y = 3.79x − 14.12	0.9980	2.12	7.08
4-Hydroxybenzoic acid	5–500	y = 7.62x + 22.79	0.9996	1.72	5.72
(−)-Epicatechin	5–500	y = 9.11x − 9.99	0.9971	1.85	6.18
Caffeic acid	5–500	y = 11.09x + 16.73	0.9997	3.15	10.50
Syringic acid	10–500	y = 0.74x − 1.54	0.9975	3.75	12.50
3-Hydroxybenzoic acid	5–500	y = 3.69x − 12.29	0.9991	1.86	6.20
Vanillin	50–500	y = 2.02x + 135.49	0.9926	15.23	50.77
Verbascoside	2.5–500	y = 8.59x − 28.05	0.9988	0.82	2.75
Taxifolin	5–500	y = 12.32x + 9.98	0.9993	1.82	6.05
Sinapic acid	5–500	y = 2.09x − 6.79	0.9974	2.64	8.78
p-Coumaric acid	5–500	y = 17.51x + 53.73	0.9997	1.93	6.44
Ferulic acid	5–500	y = 3.32x − 4.30	0.9992	1.43	4.76
Luteolin 7-glucoside	1–500	y = 45.25x + 156.48	0.9996	0.45	1.51
Hesperidin	5–500	y = 5.98x + 0.42	0.9993	1.73	5.77
Hyperoside	2.5–500	y = 16.32x − 1.26	0.9998	0.99	3.31
Rosmarinic acid	1–500	y = 9.82x − 17.98	0.9989	0.57	1.89
Apigenin 7-glucoside	1–500	y = 21.33x − 31.69	0.9983	0.41	1.35
2-Hydroxycinnamic acid	1–500	y = 16.72x − 26.94	0.9996	0.61	2.03
Pinoresinol	10–500	y = 0.80x − 2.69	0.9966	3.94	13.12
Eriodictyol	2.5–500	y = 14.24x − 0.50	0.9998	0.80	2.68
Quercetin	5–500	y = 14.68x − 18.25	0.9997	1.23	4.10
Luteolin	5–500	y = 8.96x + 26.80	0.9992	1.34	4.46
Kaempferol	10–500	y = 0.82x − 3.06	0.9959	3.30	10.99
Apigenin	2.5–500	y = 11.29x + 38.05	0.9987	0.96	3.20

LOD and LOQ: limit of detection and limit of quantification, respectively.

Table S3. Pearson correlation analysis results of methanol extract of *A. gymnolobus*

Compounds	A549-48h	HeLa-24h	HeLa-48h	MDA-MB-231-48h	MDA-MB-231-72h
Gallic acid				* p=0.02; r=0.99	
(+)-Catechin					** p=0.008; r=-0.99
p-Hydroxybenzoic acid	* p=0.05; r=-0.99	*p=0.01; r=-0.99	*p=0.05; r=-0.99		
Syringic acid					* p=0.02; r=0.98
3-Hydroxybenzoic acid				* p=0.04 r=0.98	* p=0.01 r=0.96
Ferulic acid	*p=0.04; r=0.99	*p=0.04; r=0.99	* p=0.04; r=0.99		
Hesperidin		**p=0.004; r=-0.99			
Hyperoside					* p=0.03; r=-0.96
Apigenin 7-glucoside		*p=0.05; r=0.99		* p=0.04; r=-0.99	

2. Cell proliferation (WST-1) assay

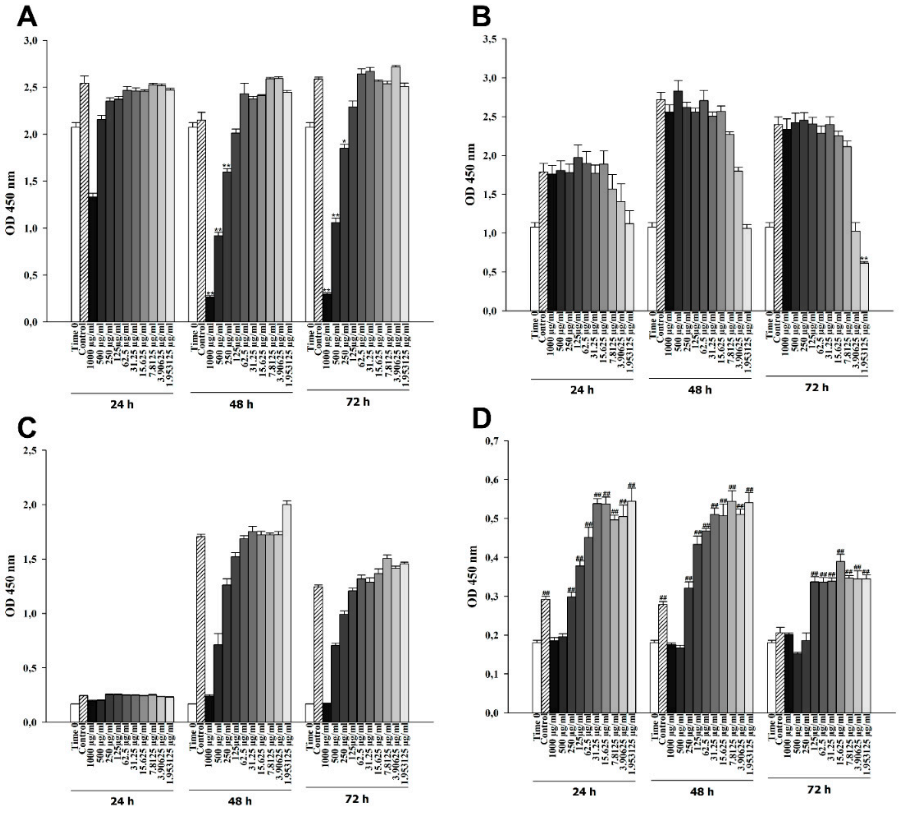

Figure S1. Effects of Fm on cell viability in vitro. Cell viability was assessed by WST-1 assays and results are presented as optical density at 450 nm (OD450) values of A549 (A), HeLa (B), MDA-MB-231 (C) and 293T (D) cells for 24, 48 and 72 h. (*P < .05, **P < .01 and ***P < .001).

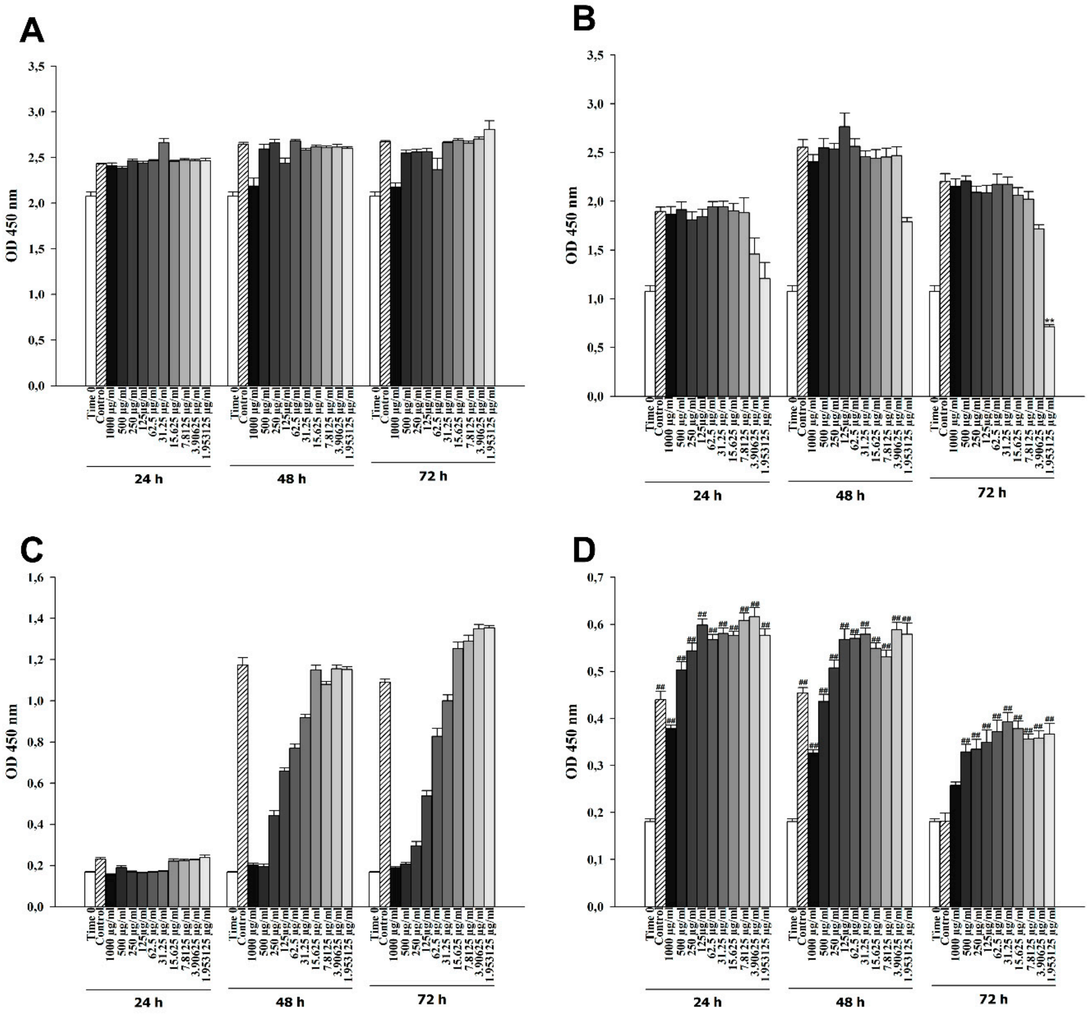

Figure S2. Effects of Lm on cell viability in vitro. Cell viability was assessed by WST-1 assays and results are presented as optical density at 450 nm (OD450) values of A549 (A), HeLa (B), MDA-MB-231 (C) and 293T (D) cells for 24, 48 and 72 h. (*P < .05, **P < .01 and ***P < .001).

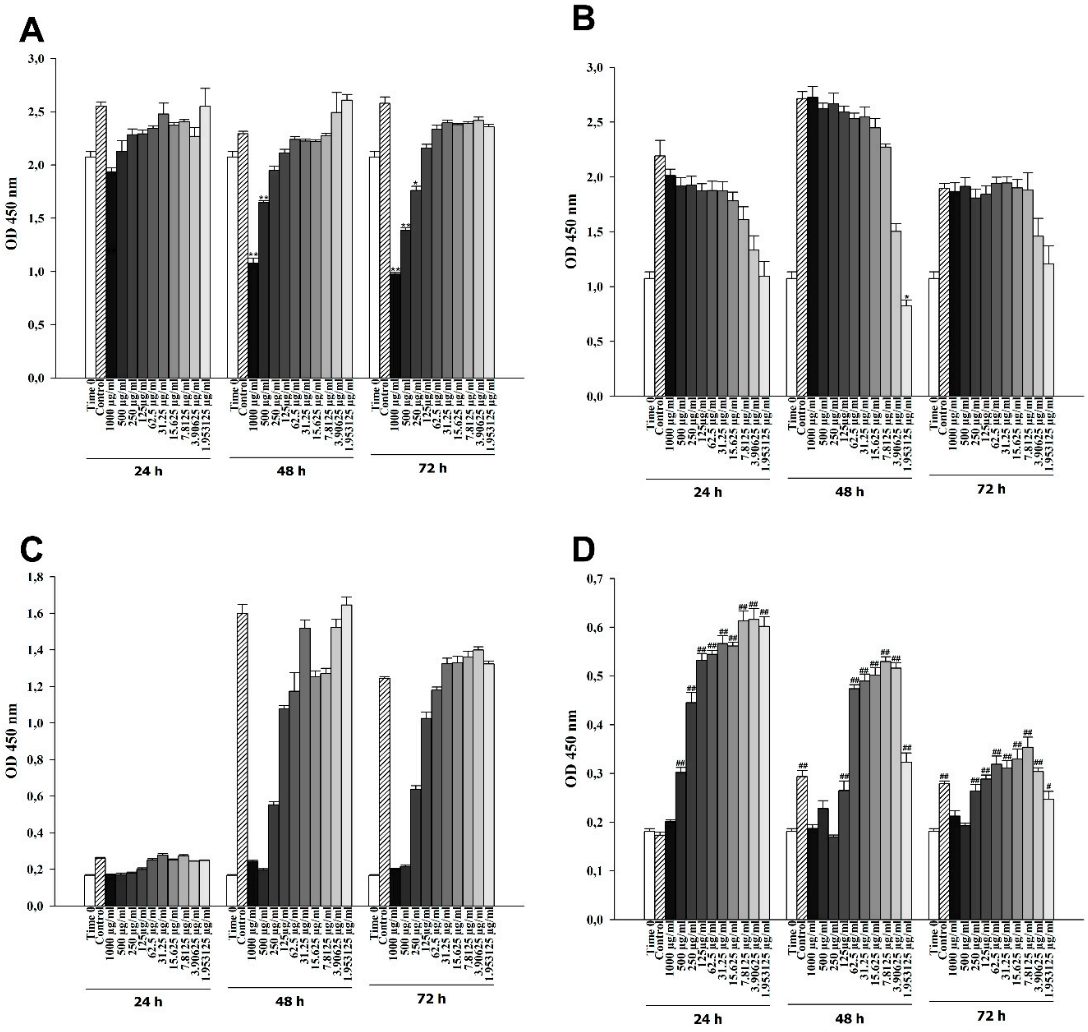

Figure S3. Effects of Rm on cell viability in vitro. Cell viability was assessed by WST-1 assays and results are presented as optical density at 450 nm (OD450) values of A549 (A), HeLa (B), MDA-MB-231 (C) and 293T (D) cells for 24, 48 and 72 h. (*P < .05, **P < .01 and ***P < .001).

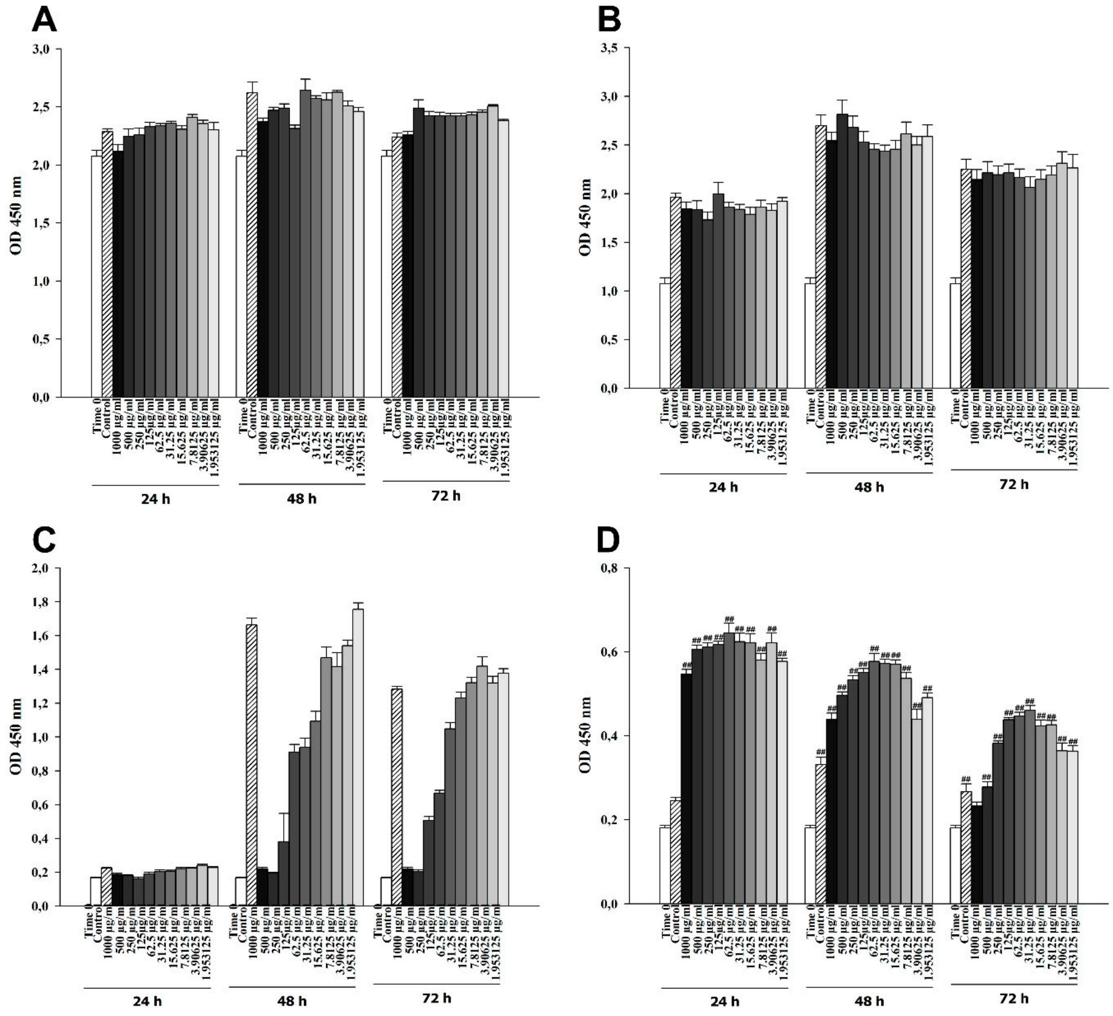

Figure S4. Effects of Sm on cell viability in vitro. Cell viability was assessed by WST-1 assays and results are presented as optical density at 450 nm (OD450) values of A549 (A), HeLa (B), MDA-MB-231 (C) and 293T (D) cells for 24, 48 and 72 h. (*P < .05, **P < .01 and ***P < .001).

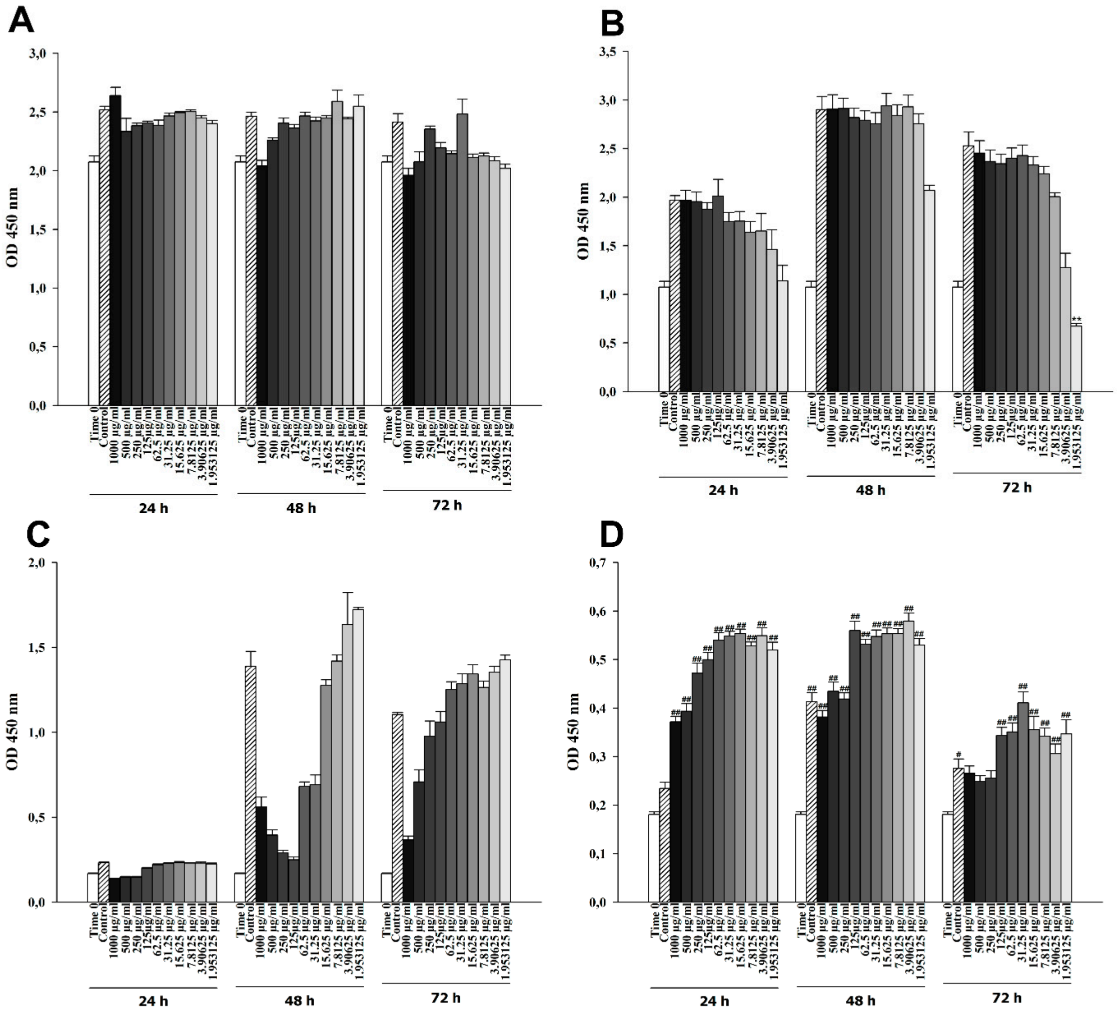

Figure S5. Effects of WPm on cell viability in vitro. Cell viability was assessed by WST-1 assays and results are presented as optical density at 450 nm (OD450) values of A549 (A), HeLa (B), MDA-MB-231 (C) and 293T (D) cells for 24, 48 and 72 h. (*P < .05, **P < .01 and ***P < .001)
